# LIFE COURSE ORIGINS OF FRAILTY IN LATER LIFE

**DOI:** 10.1093/geroni/igz038.230

**Published:** 2019-11-08

**Authors:** Monica Williams-Farrelly, Kenneth F Ferraro

**Affiliations:** Purdue University, West Lafayette, Indiana, United States

## Abstract

Frailty, generally characterized as a clinical state of increased vulnerability resulting from age-related decline in reserve and function across multiple physiologic systems, has been gaining attention in recent years due to its high correlates with a number of poor health outcomes including falls, hospitalization, and mortality. Similar to other adult health outcomes, research on the etiology of frailty has begun to move from proximal risk factors only to those more distal in time. This research uses data from the Health and Retirement study (2004-2016) to examine whether childhood exposures predict developing frailty in later life. A series of ordinal logistic regression models were estimated to test whether six domains of childhood exposures (socioeconomic status, infectious disease, chronic disease, impairments, risky adolescent behavior, and risky parental behavior) were associated with frailty, composed of five components: unintentional weight loss, weakness, slowness, exhaustion, and low energy expenditure (Fried et al., 2001). After adjusting for demographic factors, experiencing multiple SES misfortunes or risky adolescent behaviors in childhood are associated with higher odds of frailty in later life (OR= 1.24 and 2.37, respectively), while experiencing any infectious diseases is associated with lower odds of frailty (OR= 0.67 and 0.72). After further adjusting for adult characteristics, experiencing 2 or more chronic diseases in childhood is associated with a 1.35 higher odds of incident frailty over an 8-year period. These results reveal some of the early exposures that may raise frailty risk in later life but also the mid-life factors that mediate those risks.

